# Supplementary material for: Comparison between 20 and 30 meters in walkway length affecting the 6-minute walk test in patients with chronic obstructive pulmonary disease: A randomized crossover study
Source: PLoS One. 2022 Jan 7;17(1):e0262238. doi: 10.1371/journal.pone.0262238 (PMC8741022; doi:10.1371/journal.pone.0262238)
Supplement: S1 File — (PDF) [file pone.0262238.s002.pdf]

Document No. 2

Submitted protocol template

The Human Research Ethics Committee of Thammasat University. No.2

1. Project Name (Thai) การเปรียบเทียบระหว่างระยะทางเดิน 20 เมตรและ 30 เมตรที่มีผลต่อการทดสอบการเดิน 6 นาทีในผู้ป่วยโรคปอดอุดกั้นเรื้อรัง

2. Project Name (English) Comparison between 20 and 30 meters in walkway length affecting the 6-minute walk test in chronic obstructive pulmonary disease patients

3. Investigator: Mr.Apiwat Pugongchai

Office Address

Medical Diagnostics Unit, Thammasat University Hospital

Telephone: +6692-554-9614 **E-mail:** pu.apiwat@hotmail.com

4. Name of Co- Investigator

4.1 Mr.Kannakorn Intharakham

Office Address

Medical Diagnostics Unit, Thammasat University Hospital

Telephone: +6694-269-2524 **E-mail:** intharakham@hotmail.com

4.2 Assistant Professor Narongkorn Saiphoklang, M.D.

Office Address

Medical Diagnostics Unit, Thammasat University Hospital and Division of Pulmonary and Critical Care Medicine, Department of Internal medicine, Faculty of Medicine, Thammasat University

Telephone: +6689-690-9558 **E-mail:** M_Narongkorn@hotmail.com

5. Major Advisor

None

6. Specify the funding source and/or cooperating organization(s) :

☑ Currently funded amount....52,000.... baht

(Specify source of funding): ....Thammasat University Hospital...............

❑ Domestic funds / ❑ Foreign funds

❑ Public funds / ❑ Private funds

❑ Apply for funding from ....

amount ....baht

❑ No funds

7. Conflict of interest

The researcher are not had conflict of interate. Others staff will consent from participants.

8. Has the researcher ever attended a course on human research ethics?

- The investigator (3) passed the GCP online training (computer-based) course on Good Clinical Practice Guidelines (ICH-GCP) from Faculty of Medicine, Thammasat University, received the training on 14 December 2017.

- The co-investigator (4.1) passed the GCP online training (computer-based) course on Good Clinical Practice Guidelines (ICH-GCP) from Faculty of Medicine, Thammasat University. Received training on 13 December 2017.

- The co-investigator (4.2) passed Good Clinical Practice (GCP) 2009 and 2012, CITI program training for GCP 2015. and passed the Clinical or Experimental Related Ethics Course (2012 and 2014), Responsible conduct of research involving humans from the University of California Los Angeles, USA 2015.

9. Principles and rational

The 6-minute walk test (6MWT) is submaximal exercise that corresponds to functional activity used in daily activities. 6MWT is an effective exercise which is low cost, uncomplicated and provides a measure for evaluation of cardiopulmonary, musculoskeletal and nervous systems. In clinical practice, 6MWT is often used to evaluate patients with various diseases, especially COPD. 6MWT is a tool for evaluating physical activities, comparing pre-post treatment and predicting morbidity and mortality rates in COPD patients. A guideline of the American Thoracic Society (ATS) suggests the walkway distance should not be less than 30-m. Previous studies reported that a difference of 10 meters in the total walking distance affects the 6MWT result. In contrast, another study found that course length had no significant effect on walking distance 50 feet (15.24 m) to 164 feet (49.99 m). However, there are few published data on 6MWT in the COPD patient population. The aim of this study was to measure the difference of walking distance and other effects in walkways of 20-m and 30-m

10. Objectives:

To compare between the distances of 20 and 30 m long corridor affecting 6MWT in COPD patients

11. Literature Review

1) Chronic obstructive pulmonary disease, COPD

Chronic obstructive pulmonary disease is a permanently obstruction of the lung. This disease like emphysema and/or chronic bronchitis. Common symptoms include a chronic cough, dyspnea, more sputum secretion, lung wheezing, and chest tightness. It's caused by exposure to tobacco smoke, occupational chemicals, indoor and outdoor air pollution. Clinical diagnosis that should be based on carefully history taking, the presence of symptoms and assessment of airway obstruction by spirometry. The steps of the test are fully breath in and quickly and strongly breath out in one second and continuous breath long time. The last step is fully breath in again. If ratio of FEV1/FVC is less than 70%, it is diagnosed chronic obstructive pulmonary disease. This value can divide of severity of COPD. In addition, 6MWT can evaluate before and after treatment and can plan to treatment.

2) 6-minute walk test

The 6-minute walk test (6MWT) is submaximal exercise that corresponds to functional activity used in daily activities. 6MWT is an effective exercise which is low cost, uncomplicated and provides a measure for evaluation of cardiopulmonary, musculoskeletal and nervous systems. In clinical practice, 6MWT is often used to evaluate patients with various diseases, especially COPD. 6MWT is a tool for evaluating physical activities, comparing pre-post treatment and predicting morbidity and mortality rates in COPD patients. A guideline of the American Thoracic Society (ATS), 2002 suggests the walkway distance should not be less than 30-m. Previous studies interested in comparison of 10, 20, and 30-meter distances. The result found that walkway length affects to 6MWD because the long walkway has number of return less than short walkway which is consistent with study in COPD patients. These research study compared 10 and 30 meter distance. The result showed difference distance of 10 and 30 meter was 49.5 meters. In contrast, another study found that course length had no significant effect on walking distance 50 feet (15.24 m) to 164 feet (49.99 m). However, there are few published data on 6MWT in the COPD patient population. Moreover, some previous study performed in stroke patients. The result showed significance difference of 6MWD between 10, 20, and 30 meter but the study in patient with liver cirrhosis showed no significance difference of 6MWD between 20, and 30 meter.

The aim of this study was to measure the difference of walking distance and other effects in walkways of 20-m and 30-m. We hypothesized that the results would not differ between the two distances.

References

1. ATS statement: guidelines for the six-minute walk test. American journal of respiratory and critical care medicine. 2002;166(1):111-7.

2. Ng SS, Tsang WW, Cheung TH, Chung JS, To FP, Yu PC. Walkway length, but not turning direction, determines the six-minute walk test distance in individuals with stroke. Archives of physical medicine and rehabilitation. 2011;92(5):806-11.

3. Ng SS, Yu PC, To FP, Chung JS, Cheung TH. Effect of walkway length and turning direction on the distance covered in the 6-minute walk test among adults over 50 years of age: a cross-sectional study. Physiotherapy. 2013;99(1):63-70.

4. Vogelmeier CF, Criner GJ, Martinez FJ, Anzueto A, Barnes PJ, Bourbeau J, et al. Global Strategy for the Diagnosis, Management and Prevention of Chronic Obstructive Lung Disease 2017 Report. Respirology. 2017;22(3):575-601.

5. Beekman E, Mesters I, Hendriks EJ, Klaassen MP, Gosselink R, van Schayck OC, et al. Course length of 30 metres versus 10 metres has a significant influence on six-minute walk distance in patients with COPD: an experimental crossover study. Journal of physiotherapy. 2013;59(3):169-76.

6. Sciurba F, Criner GJ, Lee SM, Mohsenifar Z, Shade D, Slivka W, et al. Six-minute walk distance in chronic obstructive pulmonary disease: reproducibility and effect of walking course layout and length. American journal of respiratory and critical care medicine. 2003;167(11):1522-7.

7. Veloso-Guedes CA, Rosalen ST, Thobias CM, Andreotti RM, Galhardo FD, Oliveira da Silva AM, et al. Validation of 20-meter corridor for the 6-minute walk test in men on liver transplantation waiting list. Transplantation proceedings. 2011;43(4):1322-4.

12. Methodology

Study Design: Randomized clinical trials

Type of research (Multiple answers possible)

☑ Quantitative research

☐ Qualitative research

☐ Others; specify...

12.1 Research grouping

The study is randomized participants divided into two groups. First group is tested in a 20 meters and 30 meters, respectively. Another group is tested in a 30meters and 20 meters, respectively.

Research design

1) The researchers recruit COPD patients who come for pulmonary function test and 6-minute walk test at the Medical Diagnostics Unit, Thammasat University Hospital

2) The researchers screen COPD patients to participate in the study follow by inclusion criteria.

3) The researchers explain research information and invite to participate in the research project.

4) The study is randomly divided into two groups. 20meters is tested before in first group. After completing the first test, the participants will be rest until returning to normal vital sign. After that will be start test in 30 meters. In second group will be start in 30 and 20 meters respectively.

5) The researcher will be follow step of the test by AASM. The participants have to walk as fast as you can but not run. If participants feel tired, they can rest and continue walking when they feel better until six minutes are complete.

6) The researcher will be collect all data such as six minute walk distance in 20 and 30 meters, vital sign, and dyspnea scale. All Data are collected in a case record form.

Fig.1 Study Process

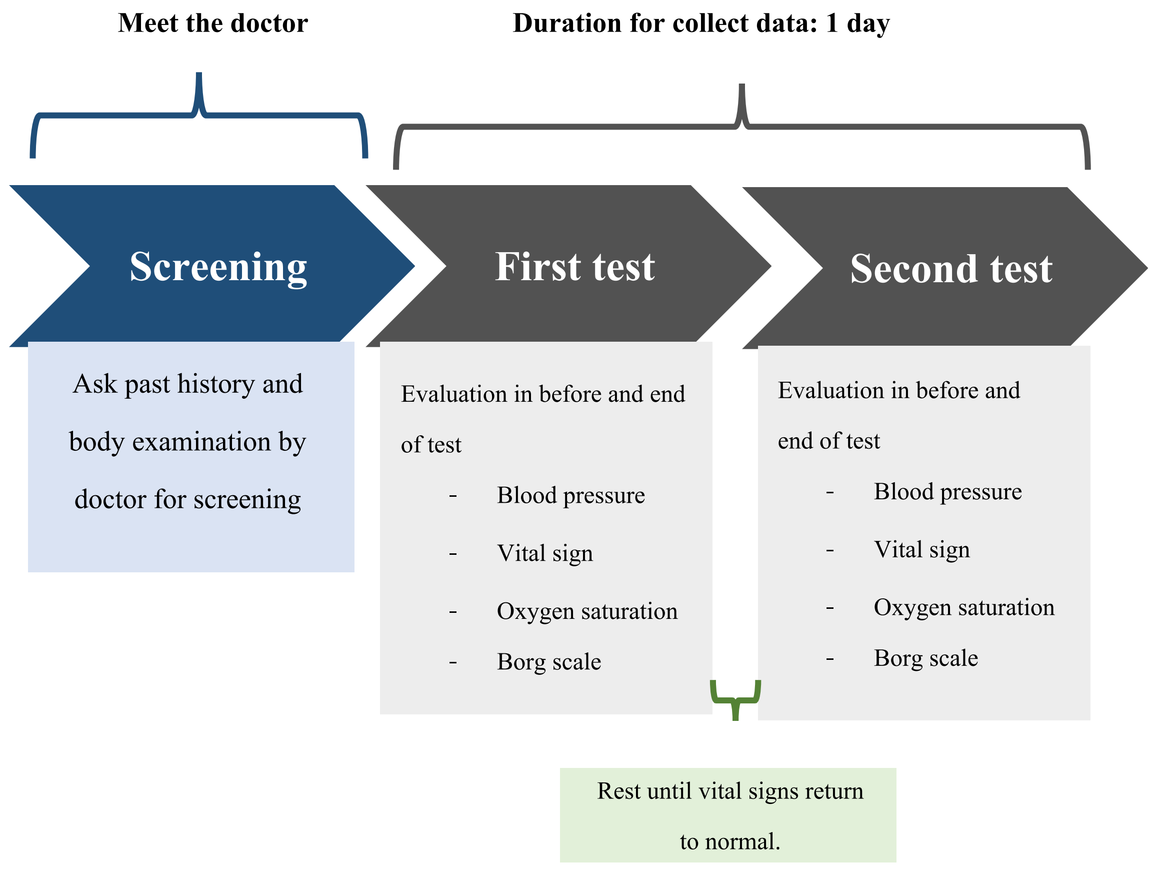

12.3 Outcome measurement

12.3.1 Measurement

This test is collected by researchers. They are scientist in the Medical Diagnostics Unit, Thammasat University Hospital

12.3.2 Tool, reliability of tools

1) Total walk distance in six minutes is measured by tape measuring. Stopwatch is used for recording times. The unit of distance is meter. The test used two cones for walk way landmark.

2) Vital sign consisted of blood pressure, heart rate, oxygen saturation, and respiratory rate.

2.1) The unit of blood pressure and heart rate are millimeter mercury (mmHg) and beat per minute (bpm) respectively. Both values are measured by Sphygmomanometer.

2.2) The unit of oxygen saturation is percentage. It is measured by pulse oximeter

2.3) The unit of respiratory rate is beat per minute. Stopwatch is used for recording time in one minute.

3) Borg dyspnea scale (Thai version) is used for evaluating level of tired. Range of this scale is from 0 to 10 (Fig.2). The result will be record in Clipboard Borg scale

Fig.2 Borg dyspnea scale (Thai version)

การประเมินความรุนแรงของความรู้สึกเหนื่อยตาม modified Borg scale

10 Point Scale (Modified Borg Scale)	
0	ไม่เหนื่อยเลย
0.5	แทบไม่เหนื่อย
1	เหนื่อยน้อยมาก
2	เหนื่อยเล็กน้อย
3	เหนื่อยปานกลาง
4	เหนื่อยค่อนข้างมาก
5	เหนื่อยมาก
6	เหนื่อยมากๆ
7	
8	
9	เหนื่อยมากเกือบที่สุด
10	เหนื่อยมากที่สุดจนทนไม่ไหว

13. Data collection

Case record form (CRF)

14. Data Analysis

- Categorical data will be show in number and percentage. Comparison between group will use chi-squared test

- Ccontinuous data will be show in mean and standard deviation. Median of comparing between group will use Student T-test or Mann Whitney U test.

- If the parameters have $p < 0.05$, it is significance.

15. Research Method

A randomized crossover study was conducted with COPD patients. The 6-minute walk test follows by the guidelines of the AASM. The participants have to walk as fast you can but not run. The researcher will be collected data before and after test such as 6MWD, vital sign, and dyspnea scale. The test performs 2 times. When the first test finished, the participant has to rest until vital sign return to normal. Then, the second test will be start. If the participants stop walking during the test, they will be withdrawing from research project. (Fig.3)

Fig.3 Research Method

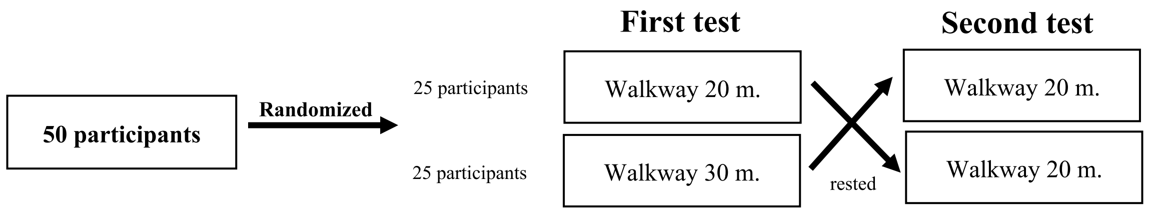

16. Target population

The participants who had diagnosed chronic obstructive pulmonary disease from specialist have results of spirometry and performed six- minute walk test in medical diagnostics Unit, Thammasat University Hospital.

17. Inclusion criteria

1. Male or female, aged 40 years or more
2. Had diagnosed chronic obstructive pulmonary disease (Spirometry show $FEV_{1/}FVC$ <70%)
3. History of smoking at least 10 pack years

18. Exclusion criteria

1. Asthma exacerbation within 6 weeks before the test
2. Myocardial infarction, eye surgery, or abdomen surgery within 6 weeks before the test
3. Other pulmonary diseases such as lung cancer, severe lung fibrosis, lung tuberculosis
4. Cannot walk or in cooperate such as dementia, neuromuscular diseases
5. Low Oxygen saturation (Lower than 90% in resting)
6. Blood pressure is lower than 90/60 mmHg or higher than 180/100 mmHg or heart rate is lower than 50 or higher than 120 beat per minute

19. Withdrawal criteria

The participants have low Oxygen saturation (less than 88%) or feel very tired, cannot continues to walk, heart palpitation, difficult to breath, leg cramp, chest pain, eye blurry. The researcher will be stop testing immediately.

20. Community Role

None

21. Sample size:

The 50 patients who had diagnosed chronic obstructive pulmonary disease from specialist

22. Sample size calculation:

The previous study in Netherlands compared walking distance of 10 and 30 meters of six minute in COPD patients. [5] The result showed that mean ± standard deviation = 49.5 ± 33.6 meters

The researcher hypothesized that difference of distance in 20 and 30 meters was 34 meters

This study used one-sample comparison of mean to hypothesized value. STATA program version 12.0 will be used in this study.

```
1 . sampsi 49.5 34, sd1(33.6) pre(0) post(1) onesample

  Estimated sample size for one-sample comparison of mean
    to hypothesized value

  Test Ho: m =     49.5, where m is the mean in the population

  Assumptions:

           alpha =    0.0500  (two-sided)
           power =    0.9000
   alternative m =        34
              sd =      33.6

  Estimated required sample size:

               n =        50
```

m = Different mean of total distance = 49.5 meters

sd = Standard deviation of difference of total distance = 33.6 meters

Alpha = Type I error = Two-sided type of statistically significance = 0.05

Power = 0.9

Alternative m = Mean difference of total distance in 20 and 30 meters expecting = 34 เมตร

Therefore, the total participants in this study are 50.

23. Consenting process

The patients who have inclusion criteria will be invited by voluntary. The researcher will explain about detail of the research. Then, the patients sign to the consent form.

24. Duration while answering the questionnaire / interview form

None

25. Number of times volunteering

1 time, 1 hour per time.

26. Total research time: 1 year (12 months)

month / Step	1	2	3	4	5	6	7	8	9	10	11	12
Project proposal and preparation	■	■										
Data collection			■	■	■	■	■	■				
Data analysis									■			
Interpret results and write reports										■		
Publication											■	■

27. Expected Benefits of the study

If the result shows no significant between distance 20 and 30 meters, the researcher will be used 20-meters distance repeating 30-meters. In contrast, If the result shows significant difference between distance 20 and 30 meters, the researcher will be used only 30 meters for 6MWT.

28. Benefits of the participants

No directly benefits to participants

29. Privacy policy

In case record form, the first-last name, hospital number (HN), address, telephone number, or other form of identification is not noted. This research use code and record electronic file in the computer that has password, other persons can't open files. In addition, CDs is collet in locker. All data is collect at Medical Diagnostics Unit, Thammasat University Hospital.

30. Duration of collection

Researchers will keep data for 1 year. After project finished, all data will be destroying.

31. How to destroy data after completing research?

Permanently delete electronic files and destroy documents of participants.

32. Risk and side effects

The participant may be tired or effect to heart activity. If participant feel very tired or uncomfortable, the researcher will be stop test immediately. After that, the participant rest until feel better. However, if participant feel not good after resting, the researcher will be contact doctor for treatment

33. Compensation or reward for participants

200 baht per person

Contract Terms:

1) The researchers sign in this document. We will conduct the research accredited by The Human Research Ethics Committee of Thammasat University. No.2 and we consent from participants rightly. The rights will be respected and the welfare of the participants is paramount.

2) I hereby certify that research study will begin after being approved by The Human Research Ethics Committee of Thammasat University. No.2.

3) If the research detail change the method, research will be notify the participants immediately.

I hereby certify that the above statement is true and understand the meaning clearly in all respects.

Signed……………………………………………

(...)

................/.................../.....................

Investigator

Signed……………………………………………

(...)

................/.................../...................

Supervisor
