# Supplementary material for: Comparison between 20 and 30 meters in walkway length affecting the 6-minute walk test in patients with chronic obstructive pulmonary disease: A randomized crossover study
Source: PLoS One. 2022 Jan 7;17(1):e0262238. doi: 10.1371/journal.pone.0262238 (PMC8741022; doi:10.1371/journal.pone.0262238)
Supplement: S2 File — (PDF) [file pone.0262238.s003.pdf]

001/2561

เอกสารหมายเลข 2

แบบเสนอโครงการวิจัยเพื่อขอรับการพิจารณารับรองจากคณะอนุกรรมการจริยธรรมการวิจัยในคน มหาวิทยาลัยธรรมศาสตร์ ชุดที่ 2

1. ชื่อโครงการวิจัย (ภาษาไทย) (Thai) การเปรียบเทียบผลการทดสอบการเดิน 6 นาที ในผู้ป่วยโรคปอดอุดกั้นเรื้อรัง ระหว่างความยาวของทางเดิน 20 เมตรกับ 30 เมตร

2. ชื่อโครงการวิจัย (ภาษาอังกฤษ) (English) Comparison between 20 and 30 meters in walkway length affecting the 6-minute walk test in chronic obstructive pulmonary disease patients

3. ชื่อผู้วิจัย (Investigator) : นายอภิวัฒน์ ภูกองไชย

สถานที่ทำงาน (Office Address)

หน่วยตรวจวินิจฉัยทางการแพทย์ โรงพยาบาลธรรมศาสตร์เฉลิมพระเกียรติ

โทรศัพท์มือถือ (Telephone) 092-5549614 **E-mail:** pu.apiwat@hotmail.com

4. ชื่อผู้ร่วมวิจัย (Name of Co- Investigator)

4.1 นายกรรณกร อินทรขำ

สถานที่ทำงาน (Office Address)

หน่วยตรวจวินิจฉัยทางการแพทย์ โรงพยาบาลธรรมศาสตร์เฉลิมพระเกียรติ

โทรศัพท์มือถือ 094-2692524 **E-mail:** intharakham@hotmail.com

4.2 ผู้ช่วยศาสตราจารย์ นายแพทย์ณรงค์กร ซ้ายโพธิ์กลาง

สถานที่ทำงาน (Office Address)

หน่วยตรวจวินิจฉัยทางการแพทย์ โรงพยาบาลธรรมศาสตร์เฉลิมพระเกียรติ และ หน่วยโรคระบบการหายใจและเวชบำบัดวิกฤต ภาควิชาอายุรศาสตร์ คณะแพทยศาสตร์ มหาวิทยาลัยธรรมศาสตร์

โทรศัพท์มือถือ 089-6909558 **E-mail:** M_Narongkorn@hotmail.com

5. ชื่ออาจารย์ที่ปรึกษาวิทยานิพนธ์หลัก (Major Advisor)

ไม่มี

คณะอนุกรรมการจริยธรรมการวิจัยในคน
มธ. ชุดที่ 2
16 มีนาคม 2561
อนุมัติ

6. **ข้อมูลทางด้านทุนวิจัย (Specify the funding source and/or cooperating organization(s) :**

ผู้วิจัยทำเครื่องหมาย ✓ ลงในช่อง ☐ ที่ตรงกับโครงการวิจัยของท่าน

☑ โครงการวิจัยนี้ได้รับทุนวิจัย (Currently funded) จำนวนเงิน 52,000 บาท

แหล่งให้ทุนวิจัย (Specify source of funding): โรงพยาบาลธรรมศาสตร์เฉลิมพระเกียรติ

☐ ทุนภายในประเทศไทย / ☐ ต่างประเทศ (Domestic or Foreign funds)

☐ ทุนรัฐบาล / ☐ เอกชน (Public or Private funds)

☐ อยู่ระหว่างการยื่นขอรับทุนจาก...

จำนวนเงิน....................................... บาท

☐ ไม่ได้รับทุนวิจัย (No funds)

7. **การมีส่วนได้ส่วนเสียในโครงการวิจัย (Conflict of interest)**

ผู้วิจัยไม่มีส่วนได้ส่วนเสียในโครงการวิจัย โดยในขั้นตอนการขอความยินยอม ผู้วิจัยจะขอให้เจ้าหน้าที่คนอื่นที่ไม่มีส่วนเกี่ยวข้องกับงานวิจัยเป็นผู้เชิญชวนเข้าร่วมวิจัย

8. **ผู้วิจัยได้เคยเข้ารับการอบรมหลักสูตรเกี่ยวกับจริยธรรมการวิจัยในคนหรือไม่**

- ผู้วิจัยผ่านการอบรมหลักสูตร GCP online training (computer-based) เรื่อง แนวทางการปฏิบัติการวิจัยทางคลินิกที่ดี (ICH-GCP) จากคณะแพทยศาสตร์ มหาวิทยาลัยธรรมศาสตร์ ได้รับการอบรมวันที่ 14 ธันวาคม 2560

- ผู้ร่วมวิจัย (4.1) ผ่านการอบรมหลักสูตร GCP online training (computer-based) เรื่อง แนวทางการปฏิบัติการวิจัยทางคลินิกที่ดี (ICH-GCP) จากคณะแพทยศาสตร์ มหาวิทยาลัยธรรมศาสตร์ ได้รับการอบรมวันที่ 13 ธันวาคม 2560

- ผู้ร่วมวิจัย (4.2) ผ่านการอบรบการปฏิบัติการวิจัยที่ดี (Good Clinical Practice; GCP) ปี พ.ศ. 2552 และ 2555, CITI program training for GCP ปี ค.ศ.2015 (พ.ศ. 2558) และผ่านการอบรบการจริยธรรมการวิจัยทางคลินิกหรือการทดลองที่เกี่ยวข้องในคน ปี พ.ศ. 2555 และ 2557, Responsible conduct of research involving human จาก University of California Los Angeles, USA ปี ค.ศ. 2015 (พ.ศ. 2558)

คณะอนุกรรมการจริยธรรมการวิจัยในคน
มธ. ชุดที่ 2
16 มีนาคม 2561
อนุมัติ

9. หลักการและเหตุผลที่ต้องทำวิจัย

การทดสอบสมรรถภาพปอดมีหลายวิธี ในปัจจุบันการทดสอบการเดิน 6 นาที (6-minute walk test) เป็นวิธีที่มีการยอมรับถึงประสิทธิภาพ ทำได้ง่ายไม่ยุ่งยาก และมีความสะดวกในการใช้งานเนื่องจากการทดสอบนี้เป็นการทดสอบด้วยการออกกำลังกายที่มีความหนักระดับเดียวกับการทำกิจวัตรประจำวัน จึงสามารถนำไปประเมินความสามารถในการทำงานของร่างกายและบอกถึงขีดจำกัดในการทำงานที่แสดงถึงการตอบสนองของระบบหายใจ ระบบหัวใจ ระบบหลอดเลือด ระบบประสาทและระบบการทำงานของกล้ามเนื้อ ดังนั้น ในทางเวชปฏิบัติมักนำมาใช้ประเมินผู้ป่วยโรคต่างๆ โดยเฉพาะโรคปอดและโรคหัวใจ เช่น การใช้ประเมินเปรียบเทียบผลก่อนและหลังการรักษาโรค การพยากรณ์ความทุพพลภาพและอัตราตาย เป็นต้น[1]

การทดสอบนี้จะทดสอบภายในเวลาที่กำหนดคือ 6 นาทีโดยผู้ถูกทดสอบเป็นผู้กำหนดความเร็วในการเดินด้วยตนเอง แล้ววัดระยะทางทั้งหมดที่ได้จากการเดิน สำหรับสถานที่ใช้ทดสอบต้องมีระยะทางตรงยาว เป็นพื้นราบแข็ง ไม่มีสิ่งกีดขวาง มีจุดกำหนดระยะทางเป็นเมตรเป็นระยะ ทั้งนี้ตามแนวปฏิบัติของสมาคมแพทย์โรคทรวงอกแห่งสหรัฐอเมริกา (American Thoracic Society) ค.ศ.2002 แนะนำว่า ต้องมีระยะทางเดินอย่างน้อย 30 เมตรในทางยาวและมีทางกว้างพอในการเดินวนกลับ[1] และการศึกษาก่อนหน้านี้พบว่าระยะทางเดิน 10 เมตรมีผลต่อระยะทางทั้งหมดที่ได้จากการเดิน[2, 3] โดยในทางเวชปฏิบัติในปัจจุบัน ทางหน่วยตรวจวินิจฉัยทางการแพทย์ โรงพยาบาลธรรมศาสตร์เฉลิมพระเกียรติ ได้ใช้ระยะทาง 12 เมตรในการทดสอบ (เนื่องจากข้อจำกัดเรื่องสถานที่) ซึ่งเป็นระยะทางการเดินที่สั้นกว่าตามแนวปฏิบัติมาตรฐานกำหนดระยะทางเดินที่ 30 เมตร โดยอาจทำให้ผลการทดสอบไม่น่าเชื่อถือ แต่รายงานการศึกษาก่อนหน้านี้พบว่าระยะทางเดิน 50 ฟุต (15.24 เมตร) ถึง 164 ฟุต (49.99 เมตร) ไม่มีความแตกต่างกันของผลการทดสอบอย่างมีนัยสำคัญ[4] ดังนั้นผู้วิจัยจึงเลือกระยะทางเดิน 20 เมตรเพื่อเปรียบเทียบกับระยะทางเดินตามมาตรฐานโดยวางแผนว่าจะนำผลการวิจัยมาประยุกต์ใช้ในสถานที่ที่มีพื้นที่จำกัดต่อไป

10. วัตถุประสงค์ของการวิจัย (Objectives: List your research objectives):

เพื่อศึกษาเปรียบเทียบผลต่อการทดสอบการเดิน 6 นาทีระหว่างระยะทางเดิน 20 เมตรและ 30 เมตร ในผู้ป่วยโรคปอดอุดกั้นเรื้อรัง ได้แก่ ระยะทางทั้งหมด สัญญาณชีพ และคะแนนอาการเหนื่อย

11. การรวบรวมรายงานที่สำคัญ ๆ ที่เกี่ยวข้องกับการวิจัยที่จะกระทำครั้งนี้อย่างครบถ้วน (Literature Review)

1) โรคปอดอุดกั้นเรื้อรัง (Chronic obstructive pulmonary disease, COPD)

โรคปอดอุดกั้นเรื้อรัง เป็นภาวะที่มีการอุดกั้นในปอดอย่างถาวร (irreversible airflow obstruction) ผู้ป่วยจะมีพยาธิสภาพของถุงลมโป่งพอง (emphysema) และ/หรือ หลอดลมอักเสบเรื้อรัง (chronic

คณะอนุกรรมการจริยธรรมการวิจัยในคน
มธ. ชุดที่ 2
16 มีนาคม 2561
อนุมัติ

bronchitis) สาเหตุหลักๆ เกิดจากการสูบบุหรี่ และยังเกิดจากการได้รับมลภาวะทางอากาศ รวมทั้งทางพันธุกรรม การวินิจฉัยว่าผู้ป่วยเป็นโรคปอดอุดกั้นเรื้อรัง ต้องอาศัยประวัติของผู้ป่วย อาการที่พบ รวมทั้งการตรวจเพิ่มเติมด้วย ซึ่งหนึ่งในวิธีมาตรฐานของการวินิจฉัยโรคนี้ คือการตรวจสมรรถภาพปอด (pulmonary function test) โดยใช้เครื่อง Spirometer โดยขั้นตอนจะให้ผู้ป่วยหายใจเข้าให้เต็มที่แล้วเป่าลมหายใจออกอย่างแรงและรวดเร็วผ่านเครื่องที่รับสัญญาณแล้ววัดค่าปริมาณอากาศที่สามารถหายใจออกอย่างรวดเร็วใน 1 วินาที (Force expiratory volume 1, FEV1) เทียบกับค่าปริมาณของอากาศเมื่อหายใจออกมาทั้งหมด (Force vital capacity, FVC) ซึ่งถ้าหากค่า FEV1/FVC ได้น้อยกว่า 70% ก็จะวินิจฉัยเป็นโรคปอดอุดกั้นเรื้อรัง[5] และยังใช้ในการแบ่งระดับความรุนแรงของผู้ป่วยได้ นอกจากนั้นยังมีการตรวจประเมินสมรรถภาพของปอดโดยการออกกำลังด้วยการเดินเป็นระยะเวลา 6 นาทีเพื่อใช้ประเมินก่อนและหลังการรักษาโรค และวางแผนการรักษาอย่างเหมาะสม

2) การทดสอบการเดิน 6 นาที (6-minute walk test)

การทดสอบการเดิน 6 นาที เป็นวิธีการทดสอบด้วยการออกกำลังกายที่มีประสิทธิภาพ ทำได้ง่าย สามารถนำไปประเมินความสามารถในการทำงานของระบบต่างๆ ของร่างกายและนำมาใช้ประเมินผู้ป่วยเปรียบเทียบผลก่อนและหลังการรักษาโรคต่างๆ โดยเฉพาะโรคปอด โรคระบบหลอดเลือดและโรคหัวใจ ซึ่งจากแนวปฏิบัติของสมาคมแพทย์โรคทรวงอกแห่งสหรัฐอเมริกา (American Thoracic Society) ค.ศ.2002 แนะนำว่า ต้องมีระยะทางเดินอย่างน้อย 30 เมตรในทางยาวในการปฏิบัติ ซึ่งสนับสนุนจากการศึกษาในผู้มีอายุ 50 ปีขึ้นไป โดยเปรียบเทียบระยะทางเดิน 10 เมตร 20 เมตร และ 30 เมตร พบว่า ระยะทางเดินมีผลต่อระยะทางทั้งหมดที่ได้จากการเดินของอาสาสมัคร เนื่องจากระยะของทางเดินที่ยาวมีจำนวนการวกกลับที่น้อยกว่าระยะของทางเดินที่สั้นจึงสามารถเดินได้ระยะทางที่มากกว่า[3] ซึ่งสอดคล้องกับการศึกษาในกลุ่มผู้ป่วยโรคปอดอุดกั้นเรื้อรังโดยเปรียบเทียบระยะของทางเดิน 10 เมตร และ 30 เมตรพบว่า ค่าเฉลี่ยระยะทางทั้งหมดที่ได้จากการเดินบนทางเดิน 10 เมตรน้อยกว่าทางเดิน 30 เมตร อยู่ประมาณ 49.50 เมตร[6] อย่างไรก็ตามยังมีข้อมูลขัดแย้งกันอยู่เนื่องจากมีการศึกษาในกลุ่มผู้ป่วยโรคปอดอุดกั้นเรื้อรังเช่นเดียวกัน พบว่าระยะของทางเดินระหว่าง 50 ฟุต (15.24 เมตร) ถึง 164 ฟุต (49.99 เมตร) ไม่มีผลต่อระยะทางทั้งหมดที่เดินได้อย่างมีนัยสำคัญ[4] นอกจากนั้นยังมีรายงานการศึกษาในผู้ป่วย 2 กลุ่ม โดยในกลุ่มแรกศึกษาในผู้ป่วยโรคหลอดเลือดสมอง (chronic stroke) เปรียบเทียบระยะทางเดิน 10 เมตร 20 เมตร และ 30 เมตร พบว่าระยะของทางเดินมีผลต่อระยะทางที่สามารถเดินได้[2] แต่ในกลุ่มที่ 2 ศึกษากลุ่มผู้ป่วยโรคตับแข็งที่รอการปลูกถ่ายเปลี่ยนตับ เปรียบเทียบระยะทางเดิน 20 เมตร และ 30 เมตร ผลที่ได้พบว่าระยะทางที่เดินได้จากทั้งสองระยะทางเดินไม่มีความแตกต่างอย่างมีนัยสำคัญ[7] ทั้งนี้ ผู้วิจัยจึงสนใจศึกษาการทดสอบการเดิน 6 นาทีในผู้ป่วยโรคปอดอุดกั้นเรื้อรังเปรียบเทียบระยะทางเดิน 20 เมตรและ 30 เมตร เพื่อนำผลที่ได้มาประยุกต์ใช้ในการตรวจบริการประจำ เพิ่มความน่าเชื่อถือของค่าที่ตรวจได้และเพิ่มประสิทธิภาพของการตรวจมากขึ้น

คณะอนุกรรมการจริยธรรมการวิจัยในคน
มธ. ชุดที่ 2
16 มีนาคม 2561
อนุมัติ

เอกสารอ้างอิง

1. ATS statement: guidelines for the six-minute walk test. American journal of respiratory and critical care medicine. 2002;166(1):111-7.
2. Ng SS, Tsang WW, Cheung TH, Chung JS, To FP, Yu PC. Walkway length, but not turning direction, determines the six-minute walk test distance in individuals with stroke. Archives of physical medicine and rehabilitation. 2011;92(5):806-11.
3. Ng SS, Yu PC, To FP, Chung JS, Cheung TH. Effect of walkway length and turning direction on the distance covered in the 6-minute walk test among adults over 50 years of age: a cross-sectional study. Physiotherapy. 2013;99(1):63-70.
4. Sciurba F, Criner GJ, Lee SM, Mohsenifar Z, Shade D, Slivka W, et al. Six-minute walk distance in chronic obstructive pulmonary disease: reproducibility and effect of walking course layout and length. American journal of respiratory and critical care medicine. 2003;167(11):1522-7.
5. Vogelmeier CF, Criner GJ, Martinez FJ, Anzueto A, Barnes PJ, Bourbeau J, et al. Global Strategy for the Diagnosis, Management and Prevention of Chronic Obstructive Lung Disease 2017 Report. Respirology. 2017;22(3):575-601.
6. Beekman E, Mesters I, Hendriks EJ, Klaassen MP, Gosselink R, van Schayck OC, et al. Course length of 30 metres versus 10 metres has a significant influence on six-minute walk distance in patients with COPD: an experimental crossover study. Journal of physiotherapy. 2013;59(3):169-76.
7. Veloso-Guedes CA, Rosalen ST, Thobias CM, Andreotti RM, Galhardo FD, Oliveira da Silva AM, et al. Validation of 20-meter corridor for the 6-minute walk test in men on liver transplantation waiting list. Transplantation proceedings. 2011;43(4):1322-4.

12. ระเบียบวิธีวิจัย (Methodology)

การออกแบบการวิจัย

Study Design: Randomized clinical trials

Type of research (Multiple answers possible)

☑ การวิจัยเชิงปริมาณ (Quantitative research)

☐ การวิจัยเชิงคุณภาพ (Qualitative research)

☐ อื่น ๆ โปรดระบุ (Others; specify)..

คณะอนุกรรมการจริยธรรมการวิจัยในคน
มธ. ชุดที่ 2
16 มีนาคม 2561
อนุมัติ

12.1 การแบ่งกลุ่มเพื่อทำการวิจัย (Research grouping)

การศึกษานี้ไม่มีการแบ่งกลุ่มเพื่อศึกษา แต่จะให้อาสาสมัครผู้เข้าร่วมการศึกษาทุกคนทำการทดสอบ 2 ครั้ง คือ ครั้งที่หนึ่งจะเดินบนระยะทาง 20 เมตร และครั้งที่สองจะเดินบนระยะทาง 30 เมตร

12.2 กรรมวิธีการศึกษาหรือดำเนินงานการวิจัย (Research design)

1) คัดกรองผู้ป่วยโรคปอดอุดกั้นเรื้อรังที่มารับบริการตรวจสมรรถภาพปอดและทดสอบการเดิน 6 นาทีที่หน่วยตรวจวินิจฉัยทางการแพทย์ โรงพยาบาลธรรมศาสตร์เฉลิมพระเกียรติ
2) คัดเลือกผู้ป่วยที่จะเข้าร่วมการศึกษาตามเกณฑ์การคัดเลือกผู้ป่วยเข้าร่วมการศึกษา
3) อธิบายชี้แจงและขอความยินยอมของผู้ป่วยเข้าร่วมโครงการวิจัยเป็นอาสาสมัคร พร้อมลงลายมือชื่อเป็นหลักฐาน
4) การทดสอบการเดิน 6 นาทีแบ่งเป็น 2 ครั้ง ในครั้งแรกจะให้อาสาสมัครทดสอบการเดินที่มีระยะทางเดิน 20 เมตร หลังจากเสร็จการทดสอบครั้งแรก จะให้อาสาสมัครนั่งพักกระทั่งหายเหนื่อยและสัญญาณชีพกลับมาปกติอยู่ในสภาวะพัก แล้วจึงทดสอบในการเดินครั้งที่ 2 ซึ่งจะเดินบนระยะทางเดิน 30 เมตร จนครบกำหนดเวลา 6 นาที
5) ผู้วิจัยจะทดสอบตามแนวปฏิบัติของสมาคมแพทย์โรคทรวงอกแห่งสหรัฐอเมริกา โดยชี้แจงอาสาสมัครจะต้องเดินเร็วเท่าที่จะทำได้ แต่ไม่วิ่ง หากอาสาสมัครมีอาการเหนื่อยจนเดินไม่ไหวสามารถจะหยุดทดสอบได้ทันที
6) เก็บข้อมูลต่างๆ ทั้งก่อนและหลังที่วัดได้จากการทดสอบการเดิน ได้แก่ ระยะทางทั้งหมดที่ได้จากการเดิน การเปลี่ยนแปลงของสัญญาณชีพ ระดับค่าออกซิเจนในร่างกายที่วัดจาก Pulse oximeter และคะแนนของการเหนื่อย (Borg dyspnea scale) ข้อมูลจะถูกเก็บในแบบบันทึกการเก็บข้อมูล (case record form)

คณะอนุกรรมการจริยธรรมการวิจัยในคน
มธ. ชุดที่ 2
16 มีนาคม 2561
อนุมัติ

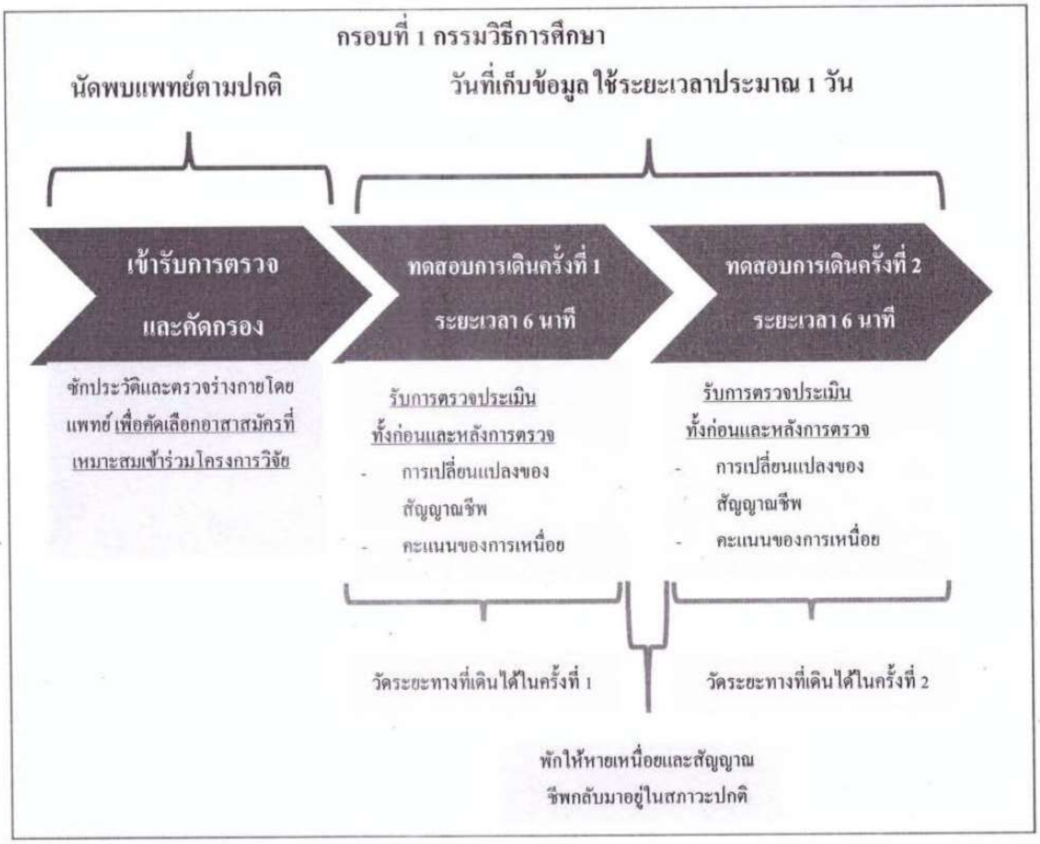

12.3 การวัดผลของการวิจัย (Outcome measurement)

12.3.1 ผู้ทำการวัด

การทดสอบและการวัดผลจะปฏิบัติโดยผู้วิจัย ซึ่งเป็นนักวิทยาศาสตร์ประจำหน่วยตรวจวินิจฉัยทางการแพทย์ โรงพยาบาลธรรมศาสตร์เฉลิมพระเกียรติ

12.3.2 เครื่องมือ, วิธีการวัด และการแปลผล

1) การวัดระยะทางทั้งหมดที่สามารถเดินได้ โดยใช้นาฬิกาจับเวลาเป็นระยะเวลา 6 นาที มีหน่วยเป็นเมตร วัดได้จากสายวัดความยาวมาตรฐาน และใช้กรวย 2 อันวางไว้เพื่อเป็นเครื่องหมายระยะห่างทุกๆ 1 เมตร เพื่อใช้ในการนับระยะทางที่เดินได้

2) สัญญาณชีพ ซึ่งประกอบไปด้วย ความดันโลหิต อัตราการเต้นของหัวใจ ระดับออกซิเจนในร่างกาย และอัตราหายใจ

คณะอนุกรรมการจริยธรรมการวิจัยในคน
มธ. ชุดที่ 2
16 มีนาคม 2561
อนุมัติ

2.1) ความดันโลหิต มีหน่วยเป็น mmHg และอัตราการเต้นของหัวใจ มีหน่วยเป็นครั้งต่อนาที ทั้ง 2 ค่า วัดได้จากเครื่องวัดความดัน (Sphygmomanometer)

2.2) ระดับออกซิเจนในร่างกาย (Oxygen saturation) มีหน่วยเป็นเปอร์เซ็นต์ (%) วัดโดยใช้เครื่องวัดระดับออกซิเจนในร่างกายที่ปลายนิ้วมือ (pulse oximeter)

2.3) อัตราหายใจ มีหน่วยเป็นครั้งต่อนาที โดยใช้นาฬิกาจับเวลาเป็นระยะเวลา 1 นาที

3) คะแนนอาการเหนื่อย (dyspnea score) วัดโดย Borg dyspnea scale มีค่าตั้งแต่ 0 ถึง 10 (ไม่เหนื่อยถึงเหนื่อยมากที่สุด) (กรอบที่ 2) ค่าที่ได้นำมาจากการสอบถามและประเมินอาการจากอาสาสมัครและบันทึกผลลงในใบบันทึกผล Clipboard Borg scale

กรอบที่ 2 คะแนนของการเหนื่อย (Borg dyspnea scale)

การประเมินความรุนแรงของความรู้สึกเหนื่อยตาม modified Borg scale

10 Point Scale (Modified Borg Scale)	
0	ไม่เหนื่อยเลย
0.5	แทบไม่เหนื่อย
1	เหนื่อยน้อยมาก
2	เหนื่อยเล็กน้อย
3	เหนื่อยปานกลาง
4	เหนื่อยค่อนข้างมาก
5	เหนื่อยมาก
6	เหนื่อยมากๆ
7	
8	
9	เหนื่อยมากเกือบที่สุด
10	เหนื่อยมากที่สุดจนทนไม่ไหว

13. การเก็บรวบรวมข้อมูล (Data collection)

เก็บข้อมูลลงในแบบบันทึกการเก็บข้อมูล (Case record form; CRF)

คณะอนุกรรมการจริยธรรมการวิจัยในคน
มธ. ชุดที่ 2
16 มีนาคม 2561
อนุมัติ

14. การวิเคราะห์ผลการวิจัย (Data Analysis)

- ข้อมูลพื้นฐานประชากร จะถูกแสดงผลเป็นจำนวนและร้อยละ การเปรียบเทียบข้อมูลระหว่างกลุ่มจะใช้สถิติ chi-squared test หรือ Fisher's Exact Test
- ระยะทางทั้งหมด สัญญาณชีพ และคะแนนอาการเหนื่อย จะถูกแสดงผลเป็นค่าเฉลี่ยและค่าเบี่ยงเบนมาตรฐาน ค่ามัธยฐานหรือสัดส่วน การเปรียบเทียบระหว่างกลุ่มจะใช้สถิติ Paired t-test หรือ Wilcoxon Rank sum Test
- ข้อมูลที่ถูกวิเคราะห์ถ้าตัวแปรใดมีค่า $p < 0.05$ ถือว่านัยสำคัญทางสถิติ

15. ขั้นตอนการวิจัย

การทดสอบการเดิน 6 นาทีจะมีขั้นตอนตามแนวปฏิบัติของสมาคมแพทย์โรคทรวงอกแห่งสหรัฐอเมริกา โดยอาสาสมัครจะต้องเดินเร็วเท่าที่จะทำได้ แต่ไม่วิ่ง โดยจะมีการเก็บข้อมูลต่างๆทั้งก่อนและหลังที่วัดได้จากการทดสอบการเดิน ได้แก่ ระยะทางทั้งหมดที่ได้จากการเดิน การเปลี่ยนแปลงของสัญญาณชีพ ระดับค่าออกซิเจนในร่างกายที่วัดจาก Pulse oximeter และคะแนนของการเหนื่อย (Borg dyspnea scale) ซึ่งในการวิจัยนี้แบ่งการทดสอบเป็น 2 ครั้ง ในครั้งแรกจะให้อาสาสมัครทดสอบการเดินที่มีระยะทางเดิน 20 เมตร หลังจากเสร็จการทดสอบครั้งแรก จะให้อาสาสมัครนั่งพักกระทั่งหายเหนื่อยและสัญญาณชีพกลับมาปกติอยู่ในสภาวะพัก แล้วจึงทดสอบในการเดินครั้งที่ 2 ซึ่งจะเดินบนระยะทางเดิน 30 เมตร หลังจากครบกำหนดเวลา 6 นาที ถือว่าสิ้นสุดการทดสอบ ถ้าอาสาสมัครมีการหยุดพักหรือหยุดเดินในระหว่างการเดิน 6 นาทีจะถูกคัดออกจากการศึกษา (กรอบที่ 3)

กรอบที่ 3 ขั้นตอนการวิจัย

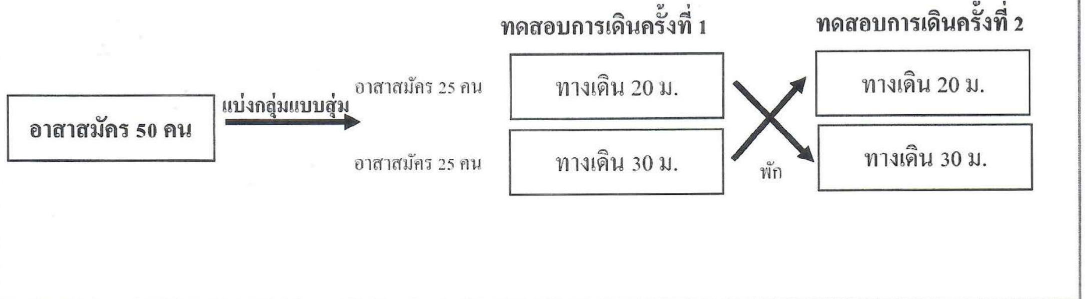

16. ผู้เข้าร่วมการวิจัยนี้คือ

อาสาสมัครผู้ป่วยโรคปอดอุดกั้นเรื้อรัง ที่ผ่านการวินิจฉัยจากแพทย์ผู้เชี่ยวชาญ ที่มารับบริการตรวจสมรรถภาพปอดและทดสอบการเดิน 6 นาทีที่หน่วยตรวจวินิจฉัยทางการแพทย์ โรงพยาบาลธรรมศาสตร์เฉลิมพระเกียรติ

คณะอนุกรรมการจริยธรรมการวิจัยในคน
มธ. ชุดที่ 2
16 มีนาคม 2561
อนุมัติ

17. เกณฑ์การคัดเลือกอาสาสมัคร (Inclusion criteria)

1. หญิงหรือชาย อายุตั้งแต่ 40-80 ปีขึ้นไป
2. ได้รับการวินิจฉัยว่าเป็นโรคปอดอุดกั้นเรื้อรัง (ผลการตรวจ spirometry มีค่า FEV_1/FVC <70%)

18. เกณฑ์การคัดออก (Exclusion criteria)

1. มีภาวะหอบกำเริบภายใน 6 สัปดาห์ก่อนเข้าการศึกษา
2. มีโรคกล้ามเนื้อหัวใจขาดเลือด ผ่าตัดตาหรือผ่าตัดในช่องท้องภายใน 6 สัปดาห์ก่อนเข้าการศึกษา
3. มีโรคปอดอื่นร่วมด้วย ได้แก่ มะเร็งปอดที่กำลังรับการรักษา ปอดเป็นผังผืดรุนแรง วัณโรคปอดระยะแพร่กระจายเชื้อ
4. ไม่สามารถเดินได้ หรือ ไม่ร่วมมือในการเดินทดสอบ เช่น โรคสมองเสื่อมรุนแรง โรคประสาทและกล้ามเนื้อ
5. ระดับออกซิเจนในร่างกายต่ำ Oxygen saturation น้อยกว่า 90% ในขณะพัก
6. ก่อนการเดินทดสอบมีภาวะความดันโลหิต ต่ำกว่า 90/60 หรือมากกว่า 180/100 มม.ปรอท อัตราเต้นหัวใจน้อยกว่า 50 หรือมากกว่า 120 ครั้งต่อนาที

19. เกณฑ์การถอนอาสาสมัครออกจากการวิจัย

1. อาสาสมัครไม่สามารถเดินครบระยะเวลา 6 นาที หรือ
2. มีระดับออกซิเจนในร่างกายต่ำ Oxygen saturation น้อยกว่า 88% หรือ
3. มีอาการเหนื่อยหอบ ใจสั่น หายใจไม่ออกรุนแรง ปวดขาเป็นตะคริวมาก เจ็บ หน้าอก เวียนศีรษะ หน้ามืด หรือตาพร่ามัว

โดยหากอาสาสมัครมีอาการดังกล่าวในขณะทดสอบ ผู้วิจัยจะให้หยุดทดสอบทันที โดยจะให้อาสาสมัครนั่งพักจนมีอาการดีขึ้น แต่หากประเมินแล้วว่าอาการไม่ดี ผู้วิจัยจะแจ้งอาจารย์แพทย์ที่ปรึกษาวิจัยหรือแพทย์เจ้าของไข้ประเมินทันทีเพื่อให้การรักษาที่เหมาะสม

20. บทบาทของชุมชน

ไม่มี

21. จำนวนกลุ่มตัวอย่าง (Sample size):

ผู้ป่วยโรคปอดอุดกั้นเรื้อรัง ที่ผ่านการวินิจฉัยจากแพทย์ผู้เชี่ยวชาญ จำนวน 50 ราย

คณะอนุกรรมการจริยธรรมการวิจัยในคน
มธ. ชุดที่ 2
16 มีนาคม 2561
อนุมัติ

22. การคำนวณขนาดตัวอย่าง (Sample size calculation):

จากการศึกษาในประเทศเนเธอร์แลนด์เรื่องการเปรียบเทียบระยะทางเดิน 10 เมตรและ 30 เมตรของการทดสอบการเดิน 6 นาทีในผู้ป่วยโรคปอดอุดกั้นเรื้อรัง[6] พบว่า ค่าเฉลี่ย ± ส่วนเบี่ยงเบนมาตรฐานของความแตกต่างของระยะทางทั้งหมดที่ได้ เท่ากับ 49.5 ± 33.6 เมตร

ผู้วิจัยตั้งสมมติฐานว่า ความแตกต่างของระยะทางทั้งหมดที่ได้จะลดลงเท่ากับ 34 เมตรเมื่อเปรียบเทียบระยะทางเดิน 20 เมตรและ 30 เมตร

การวิจัยนี้คำนวณขนาดตัวอย่างสำหรับ one-sample comparison of mean to hypothesized value โดยใช้โปรแกรม STATA version 12.0 ดังนี้

```
1 . sampsi 49.5 34, sd1(33.6) pre(0) post(1) onesample

  Estimated sample size for one-sample comparison of mean
    to hypothesized value

  Test Ho: m =     49.5, where m is the mean in the population

  Assumptions:

          alpha =    0.0500  (two-sided)
          power =    0.9000
  alternative m =        34
             sd =      33.6

  Estimated required sample size:

              n =        50
```

m = ค่าเฉลี่ยความแตกต่างของระยะทางทั้งหมดที่ได้จากการศึกษา= 49.5 เมตร

sd = ส่วนเบี่ยงเบนมาตรฐานของความแตกต่างของระยะทางทั้งหมดที่ได้จากการศึกษา = 33.6 เมตร

Alpha = Type I error = ค่าระดับนัยสำคัญทางสถิติทดสอบแบบสองทาง (two-sided) = 0.05

Power = ค่าอำนาจการทดสอบ = 0.9

Alternative m = ค่าเฉลี่ยความแตกต่างของระยะทางทั้งหมดที่ได้ระหว่าง 20 เมตรและ 30 เมตรที่คาดไว้ = 34 เมตร

ดังนั้น จำนวนขนาดตัวอย่างที่จะศึกษาอย่างน้อยเท่ากับ 50 คน

23. กระบวนการเชิญชวนให้เข้าร่วมการวิจัย

ผู้ป่วยที่มีคุณสมบัติเข้าเกณฑ์คัดเลือกอาสาสมัคร ผู้วิจัยจะขอให้เจ้าหน้าที่คนอื่นที่ไม่มีส่วนเกี่ยวข้องกับงานวิจัยเป็นผู้เชิญชวนเข้าร่วมวิจัย โดยเจ้าหน้าที่ท่านนั้นจะอธิบายเอกสารชี้แจงการวิจัย รวมถึงเอกสารขอความยินยอมให้เข้าร่วมการวิจัยให้กับผู้ป่วยและญาติได้รับทราบ โดยมีการลงนามเป็นลายลักษณ์อักษรในหนังสือยินยอมเข้าร่วมโครงการวิจัย ซึ่งหากผู้ป่วยหรือญาติต้องการทราบข้อมูลเพิ่มเติมก็สามารถสอบถามรายละเอียดได้จากผู้วิจัยโดยตรง

คณะอนุกรรมการจริยธรรมการวิจัยในคน
มธ. ชุดที่ 2
16 มีนาคม 2561
อนุมัติ

24. ระยะเวลาในขณะตอบแบบสอบถาม / แบบสัมภาษณ์ (Study duration)

ไม่มี

25. จำนวนครั้งที่นัดอาสาสมัคร

1 ครั้ง ใช้เวลาประมาณ 1 ชั่วโมง

26. ระยะเวลาในการทำวิจัยทั้งสิ้น : 1 ปี (12 เดือน)

เดือนที่ / ขั้นตอน	1	2	3	4	5	6	7	8	9	10	11	12
เสนอโครงการและเตรียมการ	■	■										
เก็บรวบรวมข้อมูล			■	■	■	■	■	■				
วิเคราะห์ข้อมูล									■			
แปลผลและเขียนรายงาน										■		
ตีพิมพ์											■	■

27. ประโยชน์ที่คาดว่าจะรับจากการวิจัย

หากผลการวิจัยพบว่า ผลระยะทางที่ผู้ป่วยเดินได้บนระยะทาง 20 เมตร ไม่แตกต่างจากระยะทาง 30 เมตร ผู้วิจัยและหน่วยงานฯ จะสามารถนำไปประยุกต์ใช้ในการจัดหาระยะทาง 20 เมตรเพื่อใช้ในการทดสอบ แต่ในทางตรงกันข้าม หากผลมีความแตกต่างกันอย่างมีนัยสำคัญ ทางหน่วยงานฯ จำเป็นต้องหาพื้นที่ที่มีระยะทาง 30 เมตรตามมาตรฐานในการทดสอบ

28. ประโยชน์ที่อาสาสมัครจะได้รับเมื่อเข้าร่วมให้ข้อมูลการวิจัย

ไม่มีประโยชน์โดยตรงต่ออาสาสมัครผู้เข้าร่วมวิจัย

29. มาตรการในการรักษาความลับของข้อมูล

ในแบบบันทึกข้อมูลของ อาสาสมัคร จะไม่มีการระบุชื่อ-นามสกุล, Hospital number (HN), ที่อยู่ เบอร์โทรศัพท์ หรือ identification รูปแบบอื่นๆ ที่สามารถระบุถึงอาสาสมัครได้เป็นรายบุคคล ซึ่งในการวิจัยจะใช้เป็นรหัสแทน โดยบันทึกเป็นไฟล์อิเล็กทรอนิกส์และเอกสาร รวมถึงบันทึกไว้ในคอมพิวเตอร์ส่วนตัวที่มีรหัสป้องกันบุคคลอื่นที่ไม่สามารถเปิดได้ และ/หรือเก็บเอกสาร, แผ่น CD ใส่ในตู้หรือลิ้นชักที่มี

คณะอนุกรรมการจริยธรรมการวิจัยในคน
มธ. ชุดที่ 2
16 มีนาคม 2561
อนุมัติ

กุญแจล็อค โดยจะเก็บข้อมูลไว้ที่หน่วยตรวจวินิจฉัยทางการแพทย์ โรงพยาบาลธรรมศาสตร์เฉลิมพระเกียรติ เพียงที่เดียว ผู้ที่สามารถเข้าถึงข้อมูลได้มีเฉพาะผู้วิจัยและผู้วิจัยร่วมเท่านั้น

30. ระยะเวลาในการเก็บรักษาข้อมูล

ผู้วิจัยจะเก็บข้อมูลไว้นาน 1 ปีหลังสิ้นสุดโครงการแล้วจะทำลายข้อมูลหลังจากนั้น

31. วิธีการทำลายข้อมูลหลังจากเสร็จสิ้นการวิจัย

ลบไฟล์อิเล็กทรอนิกส์อย่างถาวรและเผาทำลายเอกสารข้อมูลส่วนตัวของผู้ร่วมวิจัย/อาสาสมัคร

32. ความเสี่ยงที่อาจจะเกิดเหตุการณ์ไม่พึงประสงค์

อาจเกิดการหายใจเหนื่อยหอบ หรือมีผลต่อการทำงานของหัวใจ โดยหากอาสาสมัครมีอาการดังกล่าวในขณะทดสอบ ผู้วิจัยจะให้หยุดทดสอบทันที โดยจะให้อาสาสมัครนั่งพักจนมีอาการดีขึ้น แต่หากประเมินแล้วว่าอาการไม่ดี ผู้วิจัยจะแจ้งอาจารย์แพทย์ที่ปรึกษาวิจัยหรือแพทย์เจ้าของไข้ประเมินทันทีเพื่อให้การรักษาที่เหมาะสม

ทั้งนี้ ผู้วิจัยจะคำนึงถึงด้านร่างกายและภาวะโรคของผู้ป่วยระหว่างการศึกษาตลอดเวลา และพร้อมที่จะให้การดูแล สังเกตการณ์ และแก้ไขปัญหาอย่างทันถ่วงที

33. ค่าตอบแทนหรือรางวัลให้ผู้เข้าร่วมการวิจัย

อาสาสมัครที่เข้าร่วมการวิจัย จะได้รับค่าเดินทางและค่าเสียเวลาในวันเก็บข้อมูลท่านละ 200 บาท

คณะอนุกรรมการจริยธรรมการวิจัยในคน
มธ. ชุดที่ 2
16 มีนาคม 2561
อนุมัติ

ข้อสัญญา:

1) ข้าพเจ้าในฐานะผู้วิจัยได้ลงชื่อไว้ในเอกสารนี้และคณะผู้วิจัยดังมีรายนามข้างต้น จะดำเนินการวิจัยตามที่ระบุไว้ ในโครงการวิจัยฉบับที่ได้รับการรับรองจากคณะอนุกรรมการจริยธรรมการวิจัยในคน มหาวิทยาลัยธรรมศาสตร์ ชุดที่ 2 <u>และได้ขอความยินยอมจากผู้เข้าร่วมการวิจัยอย่างถูกต้องตามหลักจริยธรรมการวิจัยในคนดังที่ได้ระบุไว้ในแบบเสนอโครงการวิจัยฯ โดยจะให้ความเคารพในสิทธิและคำนึงถึงสวัสดิภาพของผู้เข้าร่วมการวิจัยเป็นสำคัญ</u>

2) ข้าพเจ้าขอรับรองว่า จะเริ่มทำการศึกษาวิจัยภายหลังจากได้รับการรับรองจากคณะอนุกรรมการจริยธรรมการวิจัยในคน มหาวิทยาลัยธรรมศาสตร์ ชุดที่ 2 เรียบร้อยแล้วเท่านั้น

3) หากมีข้อมูลที่คณะอนุกรรมการจริยธรรมการวิจัยในคน มหาวิทยาลัยธรรมศาสตร์ ชุดที่ 2 เสนอให้ปรับแก้ไข ข้าพเจ้าจะแจ้งให้ผู้ร่วมการวิจัย/คณะผู้วิจัยทราบด้วย

ข้าพเจ้าขอรับรองว่าข้อความข้างต้นเป็นความจริง และเข้าใจความหมายโดยชัดเจนทุกประการ

ลงชื่อ.....ภิรัชณ์ ภูทองไชย.....

(.....นายอภิรัชณ์ ภูทองไชย.....)

23 / 02 / 61

หัวหน้าโครงการวิจัย

ลงชื่อ.....[signature].....

ผศ.นพ.ณรงค์กร ช้ายโพธิ์กลาง

(.....หัวหน้าหน่วยตรวจวินิจฉัยทางการแพทย์)

23 / 02 / 61

ผู้บังคับบัญชา

คณะอนุกรรมการจริยธรรมการวิจัยในคน
มธ. ชุดที่ 2
16 มีนาคม 2561
อนุมัติ
