# Supplementary material for: Comparison between 20 and 30 meters in walkway length affecting the 6-minute walk test in patients with chronic obstructive pulmonary disease: A randomized crossover study
Source: PLoS One. 2022 Jan 7;17(1):e0262238. doi: 10.1371/journal.pone.0262238 (PMC8741022; doi:10.1371/journal.pone.0262238)
Supplement: S3 File — (PDF) [file pone.0262238.s004.pdf]

Document No. 4

Information Sheet

Research Project Name: Comparison between 20 and 30 meters in walkway length affecting the 6-minute walk test in chronic obstructive pulmonary disease patients

Investigator: Mr.Apiwat Pugongchai

Address: Medical Diagnostics Unit, Thammasat University Hospital

Telephone: +6692-554-9614 **E-mail:** pu.apiwat@hotmail.com

Co- Investigator

Mr.Kannakorn Intharakham

Address: Medical Diagnostics Unit, Thammasat University Hospital

Telephone: +6694-269-2524 **E-mail:** intharakham@hotmail.com

Assistant Professor Narongkorn Saiphoklang, M.D.

Address: Medical Diagnostics Unit, Thammasat University Hospital and Division of Pulmonary and Critical Care Medicine, Department of Internal Medicine, Faculty of Medicine, Thammasat University

Telephone: +6689-690-9558 **E-mail:** M_Narongkorn@hotmail.com

Dear Participant

You are invited to participate in this research project because you are diagnosed with chronic obstructive pulmonary disease (COPD). Before you decide to participate in this study, please read this document carefully to know the research's reasons and details.

You can consult your family, friends, or your doctors for participating in this research project. You have enough time to make independent decisions. If you have decided to participate in this project, please sign in the consent form.

1. Background and rational

The 6-minute walk test (6MWT) is submaximal exercise that corresponds to functional activity. This test is easy to do and provides a measure for evaluation of chronic obstructive pulmonary disease. The AASM suggests that 30-meter walkway length is an appropriate. However, the testing area in Thammasat University Hospital has not enough. Therefore, the researcher interested in a comparison between 20 and 30 meters in walkway length affecting the 6-minute walk test in chronic obstructive pulmonary disease patients

2. Objectives

To compare between the distances of 20 and 30 m long corridor affecting 6MWT in COPD patients

3. Benefits

Evidence confirms the reliability of the 6-minute walk test with a 20-meter walking distance, increases confidence in the results, and improves effective treatment.

4. The participants in the study: 50 people.

5. The duration of the research: 1 year.

6. If you decide to participate in the research, the researcher will ask you to perform a 6-minute walk test and collect data in one time.

In the study, you have to walk as fast you can but not run in six minutes. The researcher will be collected data before and after test such as 6MWD, vital sign, and dyspnea scale. The test performs 2 times. When the first test finished, you have to rest until vital sign return to normal. Then, the second test will be start. If you stop walking during the test, the researcher will stop testing immediately.

7. Research data: the case record form use code replacing first-last name. When the research finished, all data will be destroying

8. Your personal data will be kept, but it will be reported with those who involved the research and of the Human Research Ethics Committee of Thammasat University. No.2.

9. Prevention and treatment of side effects

If you are tired, you can rest until you feel better. You can continuous walking to finish the test. However, if you feel very tired, heart palpitation, difficult of breathing, leg cramp, chest pain, dizziness, or blurred vision. You can stop testing immediately.

10. Responsibilities of researcher and research sponsors when participants have complication

If you have been harmed or illness, the researcher and physician will be treated for free.

11. You will receive compensation for lost 200 baht per person.

12. If you have any questions, you can contact Mr.Apiwat Pugongchai, Mr.Kannakorn Intarakham or Assistant Professor. Dr. Narongkon Saiphoklang at Medical Diagnostics Unit, Thammasat University Hospital. Telephone 02-9269265

<u>Rights of Research Participants</u>

As a participant in a research project, you will have the following rights:

1. You will be informed background, rational, and objective of this research.
2. You will be explained about the methodology of this research.
3. You will be provided risks and uncomfortable from this research.
4. You will be explained about the benefits from this research.
5. You can ask any questions about research information or research methodology.
6. You can request to withdraw from this research any time. The withdrawal is not effect to treatment plan.
7. You will receive a copy of information sheet and consent form which has signed and consent date.
8. You can decide to participate in the research.

This research project was approved by The Human Research Ethics Committee of Thammasat University No.2. If you do not receive treatment or compensation from injury directly following in information sheet, you can contact Chair of the Human Research Ethics at Office of the Human Research Ethics Committee of Thammasat University. No.2, Research Administration Division, Office of the Rector Building, 3rd Floor, Thammasat University. Telephone/Fax 0-2564-4440-79 ext. 1804
