# Supplementary material for: Comparison between 20 and 30 meters in walkway length affecting the 6-minute walk test in patients with chronic obstructive pulmonary disease: A randomized crossover study
Source: PLoS One. 2022 Jan 7;17(1):e0262238. doi: 10.1371/journal.pone.0262238 (PMC8741022; doi:10.1371/journal.pone.0262238)
Supplement: S4 File — (PDF) [file pone.0262238.s005.pdf]

เอกสารหมายเลข 4

เอกสารชี้แจงข้อมูลแก่ผู้เข้าร่วมโครงการวิจัย

ชื่อโครงการ การเปรียบเทียบผลการทดสอบการเดิน 6 นาที ในผู้ป่วยโรคปอดอุดกั้นเรื้อรัง ระหว่างความยาวของทางเดิน 20 เมตรกับ 30 เมตร

ชื่อผู้วิจัย นายอภิวัฒน์ ภูกองไชย
ที่อยู่ หน่วยตรวจวินิจฉัยทางการแพทย์ โรงพยาบาลธรรมศาสตร์เฉลิมพระเกียรติ
โทรศัพท์มือถือ 092-5549614 **E-mail:** pu.apiwat@hotmail.com

ชื่อผู้ร่วมในโครงการวิจัย
ชื่อ นายกรรณกร อินทรขำ
ที่อยู่ หน่วยตรวจวินิจฉัยทางการแพทย์ โรงพยาบาลธรรมศาสตร์เฉลิมพระเกียรติ
โทรศัพท์มือถือ 094-2692524 **E-mail:** intharakham@hotmail.com

ชื่อ ผู้ช่วยศาสตราจารย์ นายแพทย์ณรงค์กร ซ้ายโพธิ์กลาง
ที่อยู่ หน่วยตรวจวินิจฉัยทางการแพทย์ โรงพยาบาลธรรมศาสตร์เฉลิมพระเกียรติ และ หน่วยโรคระบบการหายใจและเวชบำบัดวิกฤต ภาควิชาอายุรศาสตร์ คณะแพทยศาสตร์ มหาวิทยาลัยธรรมศาสตร์
โทรศัพท์มือถือ 089-6909558 **E-mail:** M_Narongkorn@hotmail.com

เรียน ผู้เข้าร่วมโครงการวิจัยทุกท่าน

ท่านได้รับเชิญให้เข้าร่วมในโครงการวิจัยนี้เนื่องจากท่านได้รับการวินิจฉัยเป็นโรคปอดอุดกั้นเรื้อรัง ก่อนที่ท่านจะตัดสินใจเข้าร่วมในการศึกษาวิจัยดังกล่าว ขอให้ท่านอ่านเอกสารฉบับนี้อย่างถี่ถ้วน เพื่อให้ท่านได้ทราบถึงเหตุผลและรายละเอียดของการศึกษาวิจัยในครั้งนี้

ท่านสามารถขอคำแนะนำในการเข้าร่วมโครงการวิจัยนี้จากครอบครัว เพื่อน หรือแพทย์ประจำตัวของท่านได้ ท่านมีเวลาอย่างเพียงพอในการตัดสินใจโดยอิสระ ถ้าท่านตัดสินใจแล้วว่าจะเข้าร่วมในโครงการวิจัยนี้ ขอให้ท่านลงนามในเอกสารแสดงความยินยอมของโครงการวิจัยนี้

คณะอนุกรรมการจริยธรรมการวิจัยในคน
[illegible]
16 มีนาคม 2561
อนุมัติ

1. **เหตุที่ต้องทำวิจัยและเหตุผลที่ต้องการศึกษาในคน**

เนื่องจากการทดสอบการเดิน 6 นาที เป็นการทดสอบด้วยการเดินออกกำลังกายที่มีความหนักระดับเดียวกับการทำกิจวัตรประจำวัน ทำได้ง่ายไม่ยุ่งยาก จึงสามารถนำไปประเมินความสามารถในการทำงานของระบบหายใจในผู้ป่วยโรคปอดอุดกั้นเรื้อรังได้ ในการแนะนำของระยะทางการเดินที่เหมาะสมอยู่ที่ 30 เมตร แต่ในทางเวชปฏิบัติจริง ภายในหน่วยงานไม่มีระยะทางความยาวที่เพียงพอต่อการทดสอบ จึงมีความสนใจในการวิจัยเปรียบเทียบผลต่อการทดสอบการเดิน 6 นาทีระหว่างระยะทางเดิน 20 เมตรและ 30 เมตรในผู้ป่วยโรคปอดอุดกั้นเรื้อรัง เพื่อศึกษาและนำไปประยุกต์ใช้ในอนาคต

2. **วัตถุประสงค์ของโครงการ**

   เพื่อศึกษาเปรียบเทียบผลต่อการทดสอบการเดิน 6 นาทีระหว่างระยะทางเดิน 20 เมตรและ 30 เมตรในผู้ป่วยโรคปอดอุดกั้นเรื้อรัง ได้แก่ ระยะทางทั้งหมด สัญญาณชีพ และคะแนนอาการเหนื่อย

3. **ประโยชน์ที่อาสาสมัครจะได้รับ**

   ไม่มีประโยชน์โดยตรงต่ออาสาสมัครผู้เข้าร่วมวิจัย

4. **จำนวนผู้เข้าร่วมการวิจัยทั้งสิ้น** 50 คน

5. **ระยะเวลาที่จะทำวิจัยทั้งสิ้น** 1 ปี

6. **หากท่านตัดสินใจเข้าร่วมการวิจัยแล้ว ผู้วิจัยจะขอให้ท่านมาทำการทดสอบการเดิน 6 นาทีและเก็บข้อมูลเป็นระยะเวลา 1 วัน** โดยจะทำการเดินทั้งหมด 2 ครั้ง ครั้งที่หนึ่งจะเดินบนทางเดินที่มีระยะทาง 20 เมตร หลังจากเสร็จการทดสอบครั้งที่หนึ่งแล้ว จะให้ท่านนั่งพักให้หายเหนื่อยและสัญญาณชีพกลับมาอยู่ในสภาวะปกติ จากนั้นจะไปทดสอบการเดินในครั้งที่สอง โดยจะเดินบนทางเดินที่มีระยะทาง 30 เมตร ซึ่งทั้งสองการเดิน ท่านจะต้องเดินเร็วเท่าที่จะทำได้ แต่ไม่วิ่ง นอกจากนั้นจะมีการเก็บข้อมูลต่าง ๆ ที่วัดได้จากการทดสอบการเดิน ได้แก่ ระยะทางทั้งหมดที่ได้จากการเดิน ความดันโลหิต การเปลี่ยนแปลงของสัญญาณชีพ ระดับค่าออกซิเจนในร่างกาย และคะแนนของการเหนื่อย หากท่านมีอาการเหนื่อยจนเดินไม่ไหว สามารถหยุดพักและถือว่าสิ้นสุดการทดสอบทันที

7. **ข้อมูลที่ได้จากการเก็บข้อมูล ผู้วิจัยจะใช้รหัสแทนชื่อและข้อมูลส่วนตัวของท่าน** และจะดำเนินการทำลายข้อมูลตลอดจนข้อมูลอื่นๆ ที่เกี่ยวข้องกับท่านภายหลังเสร็จสิ้นการวิจัย

   ความเสี่ยงที่อาจจะเกิดขึ้นเมื่อเข้าร่วมการวิจัย อาจเกิดการหายใจเหนื่อยหอบ หรือมีผลต่อการทำงานของหัวใจ หากท่านรู้สึกอัดอัด หรือรู้สึกไม่สบายใจ ท่านมีสิทธิ์ถอนตัวออกจากโครงการนี้เมื่อใดก็ได้ โดยไม่ต้องแจ้งให้ทราบล่วงหน้า และการไม่เข้าร่วมวิจัยหรือถอนตัวออกจากโครงการวิจัยนี้ จะไม่มีผลกระทบต่อแผนการรักษาตามปกติของท่าน

8. **ข้อมูลส่วนตัวของท่านจะถูกเก็บรักษาไว้ ไม่เปิดเผยต่อสาธารณะเป็นรายบุคคล** แต่จะรายงานผลการวิจัยเป็นข้อมูลส่วนรวม ผู้ที่มีสิทธิ์เข้าถึงข้อมูลของท่านจะมีเฉพาะผู้ที่เกี่ยวข้องกับการวิจัยนี้ และคณะอนุกรรมการจริยธรรมการวิจัยในคน มหาวิทยาลัยธรรมศาสตร์ ชุดที่ 2 เท่านั้น

คณะอนุกรรมการจริยธรรมการวิจัยในคน
มธ. ชุดที่ 2
16 มีนาคม 2561
อนุมัติ

9. **การป้องกันและการรักษาอาการข้างเคียง**

ถ้าหากท่านมีอาการเหนื่อยหอบ ใจสั่น หายใจไม่ออกรุนแรง ปวดขาเป็นตะคริวมาก เจ็บ หน้าอก เวียนศีรษะ หน้ามืด หรือตาพร่ามัว ในขณะทดสอบ ผู้วิจัยจะให้หยุดทดสอบทันที โดยจะให้อาสาสมัครนั่งพักจนมีอาการดีขึ้น แต่หากประเมินแล้วว่าอาการไม่ดี ผู้วิจัยจะแจ้งอาจารย์แพทย์ที่ปรึกษาวิจัยหรือแพทย์เจ้าของไข้ประเมินทันทีเพื่อให้การรักษาที่เหมาะสม

10. **ความรับผิดชอบของผู้ทำวิจัย/ผู้สนับสนุนการวิจัยเมื่อเกิดภาวะแทรกซ้อน**

กรณีที่ท่านได้รับอันตรายหรือเจ็บป่วยที่เป็นผลจากการศึกษา ผู้วิจัยและทีมแพทย์จะให้การดูแลรักษาท่าน โดยท่านไม่ต้องเสียค่าใช้จ่ายใดๆ ทั้งนี้ ผู้วิจัยจะคำนึงถึงด้านร่างกายและภาวะโรคของผู้ป่วยระหว่างการศึกษาตลอดเวลา และพร้อมที่จะให้การดูแล สังเกตการณ์ และแก้ไขปัญหาอย่างทันถ่วงที

11. **การวิจัยครั้งนี้ ท่านจะได้รับค่าเดินทางและค่าเสียเวลาในวันเก็บข้อมูล ท่านละ 200 บาท**

12. **หากท่านมีข้อสงสัยที่จะสอบถามเกี่ยวกับการวิจัยนี้** ท่านสามารถติดต่อไปยัง นายอภิวัฒน์ ภูกองไชย, นายกรรณกร อินทรขำ หรือ ผู้ช่วยศาสตราจารย์ นายแพทย์ณรงค์กร ซ้ายโพธิ์กลาง ได้ตลอดเวลาที่หน่วยตรวจวินิจฉัยทางการแพทย์ โรงพยาบาลธรรมศาสตร์เฉลิมพระเกียรติ เบอร์ 02-9269265

<u>**สิทธิ์ของผู้เข้าร่วมในโครงการวิจัย**</u>

ในฐานะที่ท่านเป็นผู้เข้าร่วมในโครงการวิจัย ท่านจะมีสิทธิ์ดังต่อไปนี้

1. ท่านจะได้รับทราบถึงลักษณะและวัตถุประสงค์ของการวิจัยในครั้งนี้
2. ท่านจะได้รับการอธิบายเกี่ยวกับระเบียบวิธีการของการวิจัยทางการแพทย์ที่ใช้ในการวิจัยครั้งนี้
3. ท่านจะได้รับการอธิบายถึงความเสี่ยงและความไม่สบายที่จะได้รับจากการวิจัย
4. ท่านจะได้รับการอธิบายถึงประโยชน์ที่ท่านอาจจะได้รับจากการวิจัย
5. ท่านจะมีโอกาสได้ซักถามเกี่ยวกับงานวิจัยหรือขั้นตอนที่เกี่ยวข้องกับงานวิจัย
6. ท่านจะได้รับทราบว่าการยินยอมเข้าร่วมในโครงการวิจัยนี้ ท่านสามารถขอถอนตัวจากโครงการเมื่อไรก็ได้ โดยผู้เข้าร่วมในโครงการวิจัยสามารถขอถอนตัวจากโครงการ โดยไม่ได้รับผลกระทบต่อแผนการรักษา
7. ท่านจะได้รับสำเนาเอกสารข้อมูลคำอธิบายสำหรับผู้เข้าร่วมในโครงการวิจัยและเอกสารใบยินยอมที่มีทั้งลายเซ็นและวันที่
8. ท่านมีสิทธิ์ในการตัดสินใจว่าจะเข้าร่วมในโครงการวิจัยหรือไม่ก็ได้ โดยปราศจากการใช้อิทธิพลบังคับข่มขู่ หรือการหลอกลวง

คณะอนุกรรมการจริยธรรมการวิจัยในคน
มธ. ชุดที่ 2
16 มีนาคม 2561
อนุมัติ

โครงการวิจัยนี้ได้รับการรับรองจากคณะอนุกรรมการจริยธรรมการวิจัยในคน มหาวิทยาลัยธรรมศาสตร์ ชุดที่ 2 หากท่านไม่ได้รับการรักษาพยาบาลหรือการชดเชยอันควรต่อการบาดเจ็บหรือเจ็บป่วยที่เกิดขึ้นโดยตรงจากการวิจัย หรือท่านไม่ได้รับการปฏิบัติตามที่ปรากฏในเอกสารข้อมูลคำอธิบายสำหรับผู้เข้าร่วมในการวิจัย ท่านสามารถติดต่อกับประธานคณะอนุกรรมการฯ หรือผู้แทน ได้ที่สำนักงานคณะอนุกรรมการจริยธรรมการวิจัยในคน มหาวิทยาลัยธรรมศาสตร์ ชุดที่ 2 งานวางแผนและบริหารงานวิจัย กองบริหารการวิจัย อาคารสำนักงานอธิการบดี ชั้น 3 โทรศัพท์/โทรสาร 0-2564-4440-79 ต่อ 1804

คณะอนุกรรมการจริยธรรมการวิจัยในคน
มธ. ชุดที่ 2
16 มีนาคม 2561
อนุมัติ
