# Supplementary material for: Comparison between 20 and 30 meters in walkway length affecting the 6-minute walk test in patients with chronic obstructive pulmonary disease: A randomized crossover study
Source: PLoS One. 2022 Jan 7;17(1):e0262238. doi: 10.1371/journal.pone.0262238 (PMC8741022; doi:10.1371/journal.pone.0262238)
Supplement: S5 File — (PDF) [file pone.0262238.s006.pdf]

Document No. 5

Consent Form

Research Project Name: Comparison between 20 and 30 meters in walkway length affecting the 6-minute walk test in chronic obstructive pulmonary disease patients

Date of consent...

I am (Mr./Mrs./Miss)……………………………………………………………………………………

Reading and understanding the document with researcher. The detail show that

1. Before I sign the consent form, I have (read or been informed) about rationale and objective of the project, what I will be engaged with in details, risk/ham and benefit of this project. The researcher has explained to me and I clearly understand with satisfaction.

2. The researcher certifies that I will answer any questions that I doubt willingly, without concealing or hiding until I am satisfied.

3. I participate in the research voluntarily. I can cancel this research all the time. It is not affect to treatment.

4. The researcher certifies that will keep all my information and respect privacy policy of participants. The data will be used for research and academic reasons.

5. The researcher certifies that if the research has additional information, the researcher will be notified to you immediately.

I have read the above statement and have a good understanding in all respects. and has voluntarily signed this consent form

Signed…………………………… Participant
(...)
............./.........../...........

Signed ……………………… Investigator
(...)
............./.........../...........

Signed …………………………… Witness
(...)
............./.........../...........

Signed …………………………… Witness
(...)
............./.........../...........
