# Supplementary material for: Comparison between 20 and 30 meters in walkway length affecting the 6-minute walk test in patients with chronic obstructive pulmonary disease: A randomized crossover study
Source: PLoS One. 2022 Jan 7;17(1):e0262238. doi: 10.1371/journal.pone.0262238 (PMC8741022; doi:10.1371/journal.pone.0262238)
Supplement: S6 File — (PDF) [file pone.0262238.s007.pdf]

เอกสารหมายเลข 5 001/2561

หนังสือแสดงเจตนายินยอมเข้าร่วมการวิจัย

โครงการวิจัยเรื่อง การเปรียบเทียบผลการทดสอบการเดิน 6 นาที ในผู้ป่วยโรคปอดอุดกั้นเรื้อรัง ระหว่างความยาวของทางเดิน 20 เมตรกับ 30 เมตร

วันที่ให้คำยินยอม..

ข้าพเจ้า (นาย/นาง/นางสาว)..
ขอทำหนังสือนี้ไว้ต่อหน้าหัวหน้าโครงการเพื่อเป็นหลักฐานแสดงว่า

ข้อ 1. ก่อนลงนามในหนังสือแสดงเจตนายินยอมเข้าร่วมการวิจัยนี้ ข้าพเจ้าและญาติได้รับการอธิบายจากผู้ขอความยินยอมให้ทราบถึงวัตถุประสงค์ของการวิจัย กิจกรรมการวิจัย ความเสี่ยง รวมทั้งประโยชน์ที่อาจเกิดขึ้นจากการวิจัยอย่างละเอียด และมีความเข้าใจดีแล้ว

ข้อ 2 ผู้วิจัยรับรองว่าจะตอบคำถามต่าง ๆ ที่ข้าพเจ้าและญาติสงสัยด้วยความเต็มใจ ไม่ปิดบังซ่อนเร้น จนข้าพเจ้าและญาติพอใจ

ข้อ 3 ข้าพเจ้าเข้าร่วมโครงการวิจัยนี้โดยสมัครใจ และข้าพเจ้าหรือญาติมีสิทธิที่จะบอกเลิกการเข้าร่วมในโครงการวิจัยนี้เมื่อใดก็ได้ และการบอกเลิกการเข้าร่วมวิจัยนี้จะไม่มีผลกระทบต่อแผนการรักษาที่ข้าพเจ้าจะพึงได้รับต่อไป

ข้อ 4 ผู้วิจัยรับรองว่า จะเก็บข้อมูลเฉพาะเกี่ยวกับตัวข้าพเจ้าเป็นความลับ และจะเปิดเผยได้เฉพาะในรูปที่เป็นสรุปผลการวิจัย การเปิดเผลข้อมูลเกี่ยวกับตัวข้าพเจ้าต่อหน่วยงานต่าง ๆ ที่เกี่ยวข้องกระทำได้เฉพาะกรณีจำเป็นด้วยเหตุผลทางวิชาการเท่านั้น

ข้อ 5 ผู้วิจัยรับรองว่า หากมีข้อมูลเพิ่มเติมที่ส่งผลกระทบต่อการวิจัย ข้าพเจ้าและญาติจะได้รับการแจ้งให้ทราบทันทีโดยไม่ปิดบัง ซ่อนเร้น

ข้าพเจ้าได้อ่านข้อความข้างต้นแล้วมีความเข้าใจดีทุกประการ และได้ลงนามในใบยินยอมนี้ด้วยความเต็มใจ

ลงนาม.................................ผู้ให้ความยินยอม	ลงนาม........................... ผู้ขอความยินยอม
(..)	(..)
............./.........../...........	............./.........../...........
ลงนาม.................................ญาติของผู้ให้ความยินยอม	ลงนาม.............................พยาน
(..) เกี่ยวข้องเป็น................	(..)
............./.........../...........	............./.........../...........

เวอร์ชั่นที่ 2 (แก้ไขครั้งที่ 1) วันที่ 22 กุมภาพันธ์ 2561

คณะอนุกรรมการจริยธรรมการวิจัยในคน
มธ. ชุดที่ 2
16 มีนาคม 2561
อนุมัติ
