# Supplementary material for: Comparison between 20 and 30 meters in walkway length affecting the 6-minute walk test in patients with chronic obstructive pulmonary disease: A randomized crossover study
Source: PLoS One. 2022 Jan 7;17(1):e0262238. doi: 10.1371/journal.pone.0262238 (PMC8741022; doi:10.1371/journal.pone.0262238)
Supplement: Attachment — Submitted filename: Response to Reviewers.docx [file pone.0262238.s009.docx]

Dear Reviewer of *PLOS ONE*:

I submitted an original contribution to *PLOS ONE* entitled “*Comparison between 20 and 30 meters in walkway length affecting the 6-minute walk test in patients with chronic obstructive pulmonary disease: a randomized crossover study*” (Manuscript number: PONE-D-21-27964).

Thank you so much for your great reviews and comments regarding my manuscript. I would like to explain and submit a revised manuscript. My response to the comment is below.

**RESPONSE TO REVIEWER 1:**

**REVIEWER #1 COMMENT 1:** Introduction - the parameter FEV1/FVC is not usual to express in percentage, rather is expressed in decimal number: not < 70%, but 0.70

**RESPONSE:** I have corrected this number in the Method section, Study design and participants. Thank you for kindly reviews.

**REVIEWER #1 COMMENT 2:** METHODS. It is not written which Borg scale was used, with 10 or 11 points – it should be added.

**RESPONSE:** I have added these words in the Method section, Procedures and outcomes.

**REVIEWER #1 COMMENT 3:** It is not emphasized whether patient was encouraged or not, because it is important in a methodology of 6MWT.

**RESPONSE:** I have added more information about encouragement in the Method section, Procedures and outcomes.

**REVIEWER #1 COMMENT 4:** RESULTS - In Table No 1, I suggest to delete in Legend PDE4=phosphodiesterase 4, because there was none patient with such therapy

**RESPONSE:** I have removed this word from footnote in Table 1.

**REVIEWER #1 COMMENT 5:** It is very unusual that, as we can see in table 2, the Borg Dyspnea scale was just a little above 2 points after exercise, especially because the walking distance was rather short, around 350 m, when most of COPD patients has more pronounced dyspnea...It is also unexpected that oxygen saturation did not differ before and after exercise while such short distance was achieved. Also when FEV_1_ in COPD is around 63%, we could expect longer distance in 6MWT. Please, comment that in a discussion.

**RESPONSE:** I have added more discussion in the Discussion section, Paragraph 3.

**RESPONSE TO REVIEWER 2:**

**REVIEWER #2 COMMENT 1:** The article can benefit from some minor grammatical and syntax corrections.

Example: “However, there are few published data on 6MWT in the COPD patient population” should be rephrased because there are 718 results in PubMed for 6MWT and COPD.

**RESPONSE:** I have reorganized the sentence in the Introduction section, Paragraph 2. Thank you so much for kindly reviews.

**REVIEWER #2 COMMENT 2:** The population in study (ethnicity) should be stated.

**RESPONSE:** I have added this word in the Introduction section, Paragraph 2.

**RESPONSE TO REVIEWER 3:**

**REVIEWER #3 COMMENT 1:** This is a well-designed crossover study. Randomization and sample size are clearly documented. But there is a significant problem in the introduction: the sentence is "We hypothesized that the results would not differ between the two distances." In that case, it is an equivalence study, which must be powered and analyzed completely differently. So what is it? Clearly, the sample size computations indicate a difference. Please clarify.

1. Were the parameters estimated in the sample size realized in the trial? Please add to the discussion.

**RESPONSE:** I have added more discussion about power of the test in the Discussion section, Paragraph 1. Thank you so much for wonderful reviews.

**REVIEWER #3 COMMENT 2:**

2. p<.05 has little meaning in the presence of so many hypothesis tests, as does the term "significance". Please modify the description to indicate that type I error rate is preserved for the primary outcome, and all other tests are exploratory in nature.

**RESPONSE:** I have corrected the description of secondary outcomes in the Results section, Paragraph 3. Thank you for kindly suggestions.

I appreciate your consideration of my manuscript for publication in *PLOS ONE*.

Sincerely,

Narongkorn Saiphoklang, M.D.
