# Supplementary material for: Comparison between 20 and 30 meters in walkway length affecting the 6-minute walk test in patients with chronic obstructive pulmonary disease: A randomized crossover study
Source: PLoS One. 2022 Jan 7;17(1):e0262238. doi: 10.1371/journal.pone.0262238 (PMC8741022; doi:10.1371/journal.pone.0262238)
Supplement: Attachment — Submitted filename: Response to Reviewers.docx [file pone.0262238.s010.docx]

Dear Reviewer of *PLOS ONE*:

I submitted an original contribution to *PLOS ONE* entitled “*Comparison between 20 and 30 meters in walkway length affecting the 6-minute walk test in patients with chronic obstructive pulmonary disease: a randomized crossover study*” (Manuscript number: PONE-D-21-27964).

Thank you so much for your great reviews and comments regarding my manuscript. I would like to explain and submit a revised manuscript. My response to the comment is below.

**RESPONSE TO REVIEWER:**

**REVIEWER #1:** All questions and comments by me were included as good answers and reasonable explanations, also methods are improved, so I find the manuscript ready for publishing.

**RESPONSE:** Thank you so much for wonderful reviews and accepting for my corrections.

**REVIEWER #2:** When I suggest the linguistic and semantic corrections. I mean that the whole article should be rechecked for consistence. I can still find sentences that are at least not precise like: They found that 6MWD with 30-m walkway had significantly longer than 20-m walkway in COPD patients (mean difference in 22.1 m)

That should sound like: "They found that, in COPD patients, 6MWD with 30-m walkway is significantly longer than with 20-m walkway (mean difference of 22.1 m) [10]

Please recheck the whole article and correct such sentences. I have no other comments and the article is worth publishing.

**RESPONSE:** I have corrected this sentence in the Discussion section. Thank you for kindly suggestions.

**REVIEWER #3:** The authors seem not to understand my comment on the last part of the Introduction where they say that we hypothesized that there would be NO difference. Clearly you found a difference, you powered for a difference, yet the sentence is still there. Either remove the sentence or change it to reflect that you are hypothesizing that there WILL BE a difference.

**RESPONSE:** I have removed the sentence to reflect the hypothesis in the Introduction section. Thank you for great suggestions and wonderful reviews.

I appreciate your consideration of my manuscript for publication in *PLOS ONE*.

Sincerely,

Narongkorn Saiphoklang, M.D.
